# Influence of Depressive Symptoms on Dopaminergic Treatment of Parkinson’s Disease

**DOI:** 10.3389/fneur.2014.00188

**Published:** 2014-09-25

**Authors:** Alexandru Hanganu, Clotilde Degroot, Oury Monchi, Christophe Bedetti, Béatriz Mejia-Constain, Anne-Louise Lafontaine, Sylvain Chouinard, Marie-Andrée Bruneau

**Affiliations:** ^1^Centre de Recherche, Institut Universitaire de Gériatrie de Montréal, Montreal, QC, Canada; ^2^Department of Radiology, Faculty of Medicine, University of Montreal, Montreal, QC, Canada; ^3^Centre d’Études Avancées en Médecine du Sommeil, Hôpital du Sacré Coeur de Montréal, Montreal, QC, Canada; ^4^Movement Disorders Unit, McGill University Health Center, Montreal, QC, Canada; ^5^Department of Neurology, Montreal Neurological Hospital, Montreal, QC, Canada; ^6^Unité des troubles du mouvement André Barbeau, Centre Hospitalier de l’Université de Montréal, Montreal, QC, Canada

**Keywords:** Parkinson’s disease, l-DOPA, depressive symptoms, dopamine agonists

## Abstract

**Introduction:** Depressive symptoms are very common in patients with Parkinson’s disease (PD) and have a significant impact on the quality of life. Dopaminergic medication has been shown to have an influence on the development of depressive symptoms.

**Materials and methods:** The present study analyzed two groups of non-demented patients with PD, with and without depressive symptoms, and reported the correlations between antiparkinsonian medication [specifically levodopa (l-DOPA) and dopaminergic agonists] with depressive symptoms.

**Results:** A strong statistically significant positive correlation between l-DOPA dosages and the level of depressive symptoms has been revealed, suggesting that higher l-DOPA dosages correlate with a worsening of depressive status. No significant correlation was found with dopamine agonists.

**Discussion:** The results of this study show that in patients with PD, higher l-DOPA dosages correlate with worse depressive symptoms. From this point of view, PD patients need to be better diagnosed with respect to depressive symptoms and need additional treatment adjustment when clinical manifestations of depression are present. Clinicians must be aware that dopaminergic drugs are not sufficient to alleviate depressive symptoms.

## Introduction

Parkinson’s disease (PD) is the second most frequent chronic neurodegenerative disorder, affecting up to 2% among persons older than 65 years of age (1) and nearly 10% of people older than 80 years (2). The non-motor symptoms in PD such as depression and cognitive impairment are highly prevalent and have more impact on the quality of life and health status than motor symptoms (3–5). Depression is one of the most common of these non-motor symptoms. Up to 40% of PD patients suffer from depression at the beginning of the disease [stage I on Hoehn and Yahr scale (6)] and at the stage IV, the advanced stage of the disease, up to 70% of patients will have suffered from it at some point of their illness (7–10). Additionally, depression has been considered as the single strongest predictor of quality of life in PD, even after accounting for motor functioning (11, 12).

However, depression is often under-diagnosed in PD patients (13, 14), with only 25% of patients who will actually receive effective antidepressant treatment (15, 16). This could be due to phenomenological differences between depression in PD and depression as a primary affective disorder, as it has been suggested previously (17–19). Additionally, the somatic symptoms of depression (loss of appetite, sleep disturbances, motor retardation, fatigue, loss of energy or the rarefaction of facial expressions) are very often observed in PD patients without depression and this creates an overlap between depression and Parkinsonism (20).

Previous studies brought to evidence a relationship between PD medication therapies, depressive symptoms, and cognition. Levodopa (l-DOPA) and dopaminergic agonists have been shown to reduce the depressive symptoms (21, 22), to have no effects (23, 24), or to worsen the depression in PD patients (25–28). Further, a direct correlation between depression and cognitive performance in PD patients has been emphasized (29–32) specifically with memory and executive functions (30, 33). Each episode of severe major depression in non-PD elderly people was shown to increase the risk of dementia by 14% (34) and if mild cognitive impairment was accompanied by depression, the risk of developing dementia was multiplied by two (35). This outlined the idea that depression may be a risk factor or a precursor symptom of dementia (35). Nevertheless, other studies reported no relationship between depression and cognition in PD patients (36–38), which raises the question whether medication impacts depression’s development in PD.

On the other hand, depressive symptoms have been associated with dopamine dysregulation syndrome (DDS) (39), which is an addiction-like state marked by excessive dopaminergic medication usage, particularly l-DOPA (40). PD patients with DDS have been shown to exhibit enhanced l-DOPA induced ventral striatum dopamine release compared with non-DDS patients, which led to the conclusion that sensitization of ventral striatal circuitry is associated with compulsive medication use (41). This is in line with the l-DOPA overdose in the ventral striatum due to differential dopaminergic depletion in PD (42, 43), which determines a dysregulation of ventral striatum functions. Nevertheless, a cumulative prevalence rate of DDS was not reported (40), but considering that the most common impulse control disorder present in PD, the pathological gambling, was found in 8% of patients on l-DOPA (44), the rate of DDS is probably lower.

The aim of this study was to determine the interrelation between depressive symptoms and medication, and their influence on cognition. Based on previous studies (25–28), we hypothesized that l-DOPA will have a negative impact on depressive symptoms. For this purpose, the present study explored: (a) the comparison between PD patients with depressive symptoms and those without depressive symptoms, and (b) the correlation between dopaminergic medication (l-DOPA and agonists), depressive symptoms, and cognition.

## Materials and Methods

### Subjects

Forty non-demented PD patients were included in this study. They were right and left handed, at stages I and II of the Hoehn and Yahr scale (6). All patients were recruited from the Movement Disorders Unit of the McGill University Health Center, and the Unité des Troubles du Mouvement André Barbeau, in Montreal. Patients were diagnosed by movement disorders neurologists and met the United Kingdom Brain Bank criteria for the diagnosis of idiopathic PD (45). Clinical characteristics, including medication, are presented in Table 1. All PD patients were receiving dopaminergic medication and were responsive to it. Patients with other comorbidities (e.g., stroke, Alzheimer’s disease), with history of significant brain trauma or with severe psychiatric disturbance (e.g., schizophrenia, bipolar disorder) were excluded from the study. None of the patients were taking specific antidepressant medication at the time of the study or at least 3 months prior to it. All neuropsychological procedures were conducted in accordance with the Declaration of Helsinki. All participants provided written informed consent and the protocol was approved by the Research Ethics Committee of the Regroupement Neuroimagerie Québec.

**Table 1:** **Demographic data for patients with Parkinson’s disease (PD)**.

	PD-non- depressed	PD-depressed	*P*
Number (female/male)	20 (9/11)	20 (5/15)	*p* = 0.19^c^
Age (years)^a^	60.225 ± 5.64	63.05 ± 5	*p* = 0.087
Education (years)^a^	14.05 ± 3.25	14.25 ± 3.04	*p* = 0.74
l-DOPA dosage (mg)^a^	282.5 ± 232.99	535 ± 317.93	*p* = 0.027^b^
Agonists dosage (mg)^a^	1.69 ± 1.46	2.15 ± 2.7	*p* = 0.092
UPDRS off total^a^	26.42 ± 8.12	30.45 ± 8.65	*p* = 0.079
UPDRS off tremor^a^	0.48 ± 0.40	0.53 ± 0.36	*p* = 0.058
UPDRS off rigidity^a^	1.02 ± 0.43	1.27 ± 0.45	*p* = 0.09
MoCA on^a^	28.06 ± 1.6	26.76 ± 1.72	*p* = 0.031^b^
MoCA off^a^	28.05 ± 1.23	26.53 ± 2.19	*p* = 0.035^b^
BDI total^a^	4.7 ± 2.33	14.6 ± 6.62	*p* = 0.0001^b^
BDI cognition^a^	0.64 ± 0.72	2.13 ± 1.36	*p* = 0.002^b^
BDI somatic^a^	2.89 ± 1.53	4.71 ± 2.11	*p* = 0.002^b^
BDI mood^a^	1.28 ± 1.32	5.94 ± 3.9	*p* = 0.001^b^
RAVLT-Trial 15^a^	0.24 ± 0.96	−0.97 ± 1.28	*p* = 0.054
RAVLT-Trial 6^a^	0.35 ± 0.79	−0.75 ± 1.35	*p* = 0.061
RAVLT-Trial 7^a^	0.43 ± 0.93	−0.62 ± 1	*p* = 0.021^b^
RAVLT-Recognition list A^a^	−0.03 ± 1.15	−1.87 ± 2.48	*p* = 0.031^b^
Brixton^a^	5.35 ± 2.03	5.15 ± 2.21	*p* = 0.81
Medication, no.of patients			
l-DOPA/DDCI	15	18	
Agonist	12	17	
MAO-B inhibitor	9	9	
COMT Inhibitor	6	9	

*PD-depressed, patients with Parkinson’s disease and with depressive symptoms; PD-non-depressed, patients with Parkinson’s disease and without depressive symptoms; l-DOPA, levodopa based medication; agonist, dopaminergic agonist medication; UPDRS off total/tremor/rigidity, United Parkinson’s Disease Rating Scale Part III, off-medication for the total scores, the rigidity corresponding scores and the tremor corresponding scores; MoCA on/off, Montreal Cognitive Assessment Scale, on-/off-medication; BDI, Beck Depression Inventory; cognition/somatic/mood/affect, the cognition/somatic/mood/affect sub-groups of the BDI scale; RAVLT, Rey Auditory Verbal Learning Test; Brixton, Brixton test; DDCI, DOPA decarboxylase inhibitor; MAO-B, monoamine oxidase isoform B; COMT, cathechol-*O*-methyltransferase*.

*^a^Mean scores ± SD are presented*.

*^b^This value indicates a significant difference between groups*.

*^c^Difference was assessed using the χ^2^-test*.

### Evaluation

Each PD patient underwent an evaluation of the severity of the disease using the Unified Parkinson’s Disease Rating Scale Part III (UPDRS), an assessment of the cognitive status using the Montreal Cognitive Assessment scale (MoCA) (46), and a comprehensive neuropsychological evaluation battery, performed by a licensed neuropsychologist (Dr. MCB). During the neuropsychological evaluation, the assessment of the UPDRS and the MoCA scales, all PD patients were off-medication, and did not receive any drugs related to PD for at least 12 h prior to the evaluation. A second evaluation using the MoCA scale on-medication was performed after 1 week. The neuropsychological assessment was based on the five relevant cognitive domains suggested by the Movement Disorders Society Task-Force for level II comprehensive assessment (47): attention and working memory; executive function; language; memory; visuospatial functions (Table 2). We chose the off-medication period due to several reasons: (A) previous studies reported that dopamine loss in the ventral striatum is less severe than in the putamen and dorsal striatum (48), and depression has been associated with functional activity in the ventral striatum (49, 50); (B) it has been suggested that l-DOPA doses necessary to restore the dopamine loss in the dorsal striatum may overdose the less severely depleted areas, i.e., the ventral striatum (51) thereby having the potential of inducing the depressive symptoms due to this overdose; (C) l-DOPA medication withdrawal has a detrimental effect on the cognitive loop between the dorsolateral prefrontal cortex and the dorsal caudate nucleus, specifically with task-set switching (51), thus we argue that l-DOPA treatment enhances the cognitive capacities in PD patients in this specific loop. From this point of view, studying the patients in an off-medication period, allowed us to analyze the real off-medication level of cognitive impairment, to quantify it, and to correlate it with depression symptoms.

**Table 2:** **Neuropsychological test battery according to cognitive domain**.

Cognitive domain	Test
Attention and working memory	Trail Making Test Part A (53) Digit span test (54) Stroop color-word test, reading and color naming parts (55) Tower of London (56) Brixton (57)
Executive function	MEC, orthographic verbal fluency subtest (58) Trail Making Test Part B (53) Stroop color-word test, interference part (55) Wechsler Abbreviated Scale of Intelligence, vocabulary subtest (59)
Language	Boston Naming (60) MEC, semantic verbal fluency subtest (58) Rey Auditory Verbal Learning Test (61)
Memory	Wechsler Memory Scale third edition, logical memory subtest (immediate and delayed recalls) (54) Hooper Visual Organization Test (62)
Visuospatial function	Clock-drawing subtest of the MoCA, evaluated by scores of Schulman et al.(46, 63, 64)

*MEC, Montreal evaluation of communication protocol MoCA, Montreal Cognitive Assessment Scale*.

The depressive status was evaluated using the Beck Depression Inventory II scale (BDI-II) (52) when patients were off-medication. Because the complete BDI-II evaluation is not sufficient to apply a clear diagnosis of depression, as this cannot substitute a formal clinical evaluation, we considered the intensity of depressive symptoms as an evaluation factor.

The median BDI-II score, which was 9.8, was considered the distinguishing level for creating two groups – PD-depressed (patients with a score higher than the median BDI-II and which were considered to have significant depressive symptoms) and PD-non-depressed (patients with a lower score and without significant depressive symptoms). Thus, the results of this study refer to the intensity of depressive symptoms as computed using the BDI-II scale. In order to reveal the presence of any correlation trends, we also analyzed the results for all PD patients as a group (PD-All).

Medications were grouped depending on their action. Thus, the “l-DOPA” group included the drugs that use the dopamine precursor (l-DOPA) and a DOPA decarboxylase inhibitor (such as carbidopa or benserazide) and the group of “Agonists” included the medication that activates the dopamine receptors in the absence of dopamine (ropinirole and pramipexole).

### Statistical analysis

The overall analysis was performed using the statistical software package SPSS 17 (SPSS, Chicago, IL, USA). We divided the BDI-II scale in three components: the cognitive component (comprising the items: 12. loss of interest; 13. indecisiveness; 19. concentration difficulty), the physical or “somatic” component (based on the items: 15. loss of energy; 16. changes in sleeping pattern; 18. changes in appetite; 20. tiredness; 21. loss of interest in sex), and the affective or “mood” component (comprising the items: 1. sadness; 2. pessimism; 3. past failure; 4. loss of pleasure; 5. guilty feelings; 6. punishment feelings; 7. self-dislike; 8. self-criticalness; 9. suicidal thoughts or wishes; 10. crying; 11. agitation; 14. worthlessness; 17. irritability) (65, 66). This was performed in order to analyze the significance of each component and to assess which aspects of this scale are more sensitive to antiparkinsonian therapy. Additionally, the UPDRS scale was divided into two sub-groups: the tremor sub-group, which included the five tremor related scores of the UPDRS, and the rigidity sub-group, which included the five questions related to rigidity. Analyses were performed initially for all PD patients and afterward in a separate manner for two groups: the PD-depressed and the PD-non-depressed groups. The distribution of variables of the PD groups showed a normal distribution and no outliers were found. To compare the groups on the continuous variables, an ANOVA analysis and a Pearson product–moment correlation coefficient analysis was performed. The included continuous variables were: age; daily dosage of l-DOPA; daily dosage of agonist medication; MoCA scores on-medication; MoCA scores off-medication; neuropsychological scores; components of the BDI-II scale (cognition, somatic, mood); sub-groups of the motor part of the UPDRS scale (tremor and rigidity). Categorical variables were assessed using the χ^2^-test. Statistical threshold was set to *p* < 0.05. Due to the fact that BDI components and RAVLT neuropsychological tests revealed significant results, an additional *post hoc* Bonferroni correction (67) was performed for the BDI results (which included BDI total, BDI cognition, BDI somatic, and BDI mood) and for the RAVLT results (which included RAVLT-Trial 15, RAVLT-Trial 6, RAVLT-Trial 7, and RAVLT-Recognition list A), which lowered the statistical significance to *p* < 0.0125. Bivariate analyses were performed in order to validate the correlation results and no outliers were identified.

## Results

### ANOVA results

The two PD groups did not differ in age, education, or evolution of disease (as measured by the UPDRS scale). Nevertheless, a statistically significant difference was determined between the PD-depressed and PD-non-depressed groups with respect to the daily dosage of l-DOPA (*p* = 0.027). Strikingly, the PD-depressed patients received higher dosages of l-DOPA (Table 1). Additionally, these two groups were different with respect to MoCA cognitive performance on and off-medication (*p* = 0.03) as well as the neuropsychological scores of RAVLT-Trial 7, delayed recall (*p* = 0.021), and RAVLT-recognition list A, recognition test (*p* = 0.031), with the PD-depressed group always having the worse performance. It should be noted that these differences did not remain significant after applying the *post hoc* Bonferroni correction.

### Correlations in all PD patients

The analysis for the group of all PD patients revealed a strong positive correlation between medication dosage (l-DOPA and agonists) and duration of disease, indicating that the more the duration of the disease, the higher the dosages of l-DOPA and agonists (Table 3). Furthermore, only l-DOPA dosage showed a positive correlation with the total UPDRS score, with the rigidity sub-group score of the UPDRS scale and with cognition and somatic components of the BDI-II scale. These correlations indicated that with higher UPDRS total score and higher UPDRS rigidity score, higher doses of l-DOPA were used. Importantly, a higher dosage of l-DOPA was associated with higher scores for depressive symptoms, specifically the cognition and somatic components of the BDI-II scale (to note that higher BDI scores mean worse clinical presentation of the BDI-II components of “cognition” and “somatic”). Additionally, only l-DOPA revealed negative correlation with all RAVLT scores (recognition, trial 15, trial 6, and trial 7), indicating that an increased dosage of l-DOPA might have the potential of inducing worse performance on cognitive tests like verbal learning and memory, retention of information, encoding and retrieval of information, and subjective organization. On the other hand, dopaminergic agonists showed no other correlations.

**Table 3:** **Correlation results between the groups**.

		Duration of disease	UPDRS off total	UPDRS off rigidity	BDI cognition	BDI somatic	RAVLT- recognition	RAVLT 15	RAVLT 6	RAVLT 7	Brixton
PD-All	l-DOPA	*r* = 0.423; *p* = 0.006^a^	*r* = 0.461; *p* = 0.003^a^	*r* = 0.491; *p* = 0.001^a^	*r* = 0.417; *p* = 0.018^a^	*r* = 0.353; *p* = 0.044^a^	*r* = −0.542; *p* = 0.001^b^	*r* = −0.384; *p* = 0.014^b^	*r* = −0.383; *p* = 0.015^b^	*r* = −0.408; *p* = 0.009^b^	*r* = −0.05; *p* = 0.341
	Agonists	*r* = 0.392; *p* = 0.035^a^	*r* = −0.019; *p* = 0.924	*r* = −0.01; *p* = 0.958	*r* = −0.033; *p* = 0.883	*r* = −0.035; *p* = 0.87	*r* = 0.118; *p* = 0.542	*r* = 0.037; *p* = 0.849	*r* = 0.102; *p* = 0.597	*r* = 0.043; *p* = 0.823	*r* = 0.047; *p* = 0.809
PD-non-depressed	l-DOPA	*r* = 0.607; *p* = 0.005^a^	*r* = 0.403; *p* = 0.078	*r* = 0.446; *p* = 0.049^a^	*r* = 0.445; *p* = 0.074	*r* = 0.38; *p* = 0.132	*r* = −0.394; *p* = 0.086	*r* = −0.118; *p* = 0.619	*r* = −0.028; *p* = 0.908	*r* = −0.14; *p* = 0.555	*r* = 0.425; *p* = 0.062
	Agonists	*r* = 0.588; *p* = 0.044^a^	*r* = −0.151; *p* = 0.639	*r* = −0.028; *p* = 0.93	*r* = 0.302; *p* = 0.397	*r* = 0.138; *p* = 0.703	*r* = −0.181; *p* = 0.174	*r* = 0.227; *p* = 0.478	*r* = 0.235; *p* = 0.461	*r* = 0.25; *p* = 0.434	*r* = −0.121; *p* = 0.707
PD-depressed	l-DOPA	*r* = 0.189; *p* = 0.426	*r* = 0.418; *p* = 0.067	*r* = 0.425; *p* = 0.062	*r* = 0.218; *p* = 0.649	*r* = 0.485; *p* = 0.057	*r* = −0.465; *p* = 0.039^b^	*r* = −0.288; *p* = 0.218	*r* = −0.329; *p* = 0.156	*r* = −0.34; *p* = 0.142	*r* = −0.537; *p* = 0.015^b^
	Agonists	*r* = 0.307; *p* = 0.23	*r* = 0.054; *p* = 0.836	*r* = 0.013; *p* = 0.961	*r* = −0.344; *p* = 0.249	*r* = −0.275; *p* = 0.342	*r* = 0.293; *p* = 0.253	*r* = −0.14; *p* = 0.957	*r* = 0.104; *p* = 0.69	*r* = −0.026; *p* = 0.923	*r* = 0.161; *p* = 0.537

*PD-non-depressed, patients with Parkinson’s disease and without depressive symptoms; PD-depressed, patients with Parkinson’s disease and with depressive symptoms; l-DOPA, levodopa-based medication; Agonists, dopaminergic agonist medication; UPDRS off, United Parkinson’s Disease Rating Scale, off-medication; UPDRS off rigidity, the rigidity sub-group of the UPDRS scale, which included the five questions related to rigidity; BDI, Beck Depression Inventory; cognition/somatic, the cognition/somatic sub-groups of the BDI scale; RAVLT, Rey Auditory Verbal Learning Test; Brixton, Brixton test. If *r* = ±0.7 or higher, there is a very strong positive/negative relationship; if *r* = ±0.4 to ±0.69, there is a strong positive/negative relationship; if *r* = ±0.3 to ±0.39, there is a moderate positive/negative relationship*.

*^a^This value indicates a positive correlation*.

*^b^This value indicates a negative correlation*.

### Correlations in depressed and non-depressed PD patients

The PD-non-depressed patients revealed strong positive correlations between medication dosage (l-DOPA and agonists) and duration of disease. This result was also revealed in the PD-All group, which suggests that PD-non-depressed patients where driving the positive correlation for PD-All. On the other hand, no such correlation was observed in the PD-depressed patients. Additionally, the PD-All group showed a positive correlation between l-DOPA and the rigidity sub-group score of the UPDRS scale and this same correlation was present only in PD-non-depressed patients, which confirmed that this group was driving the significant results.

It should also be noted that only PD-depressed patients revealed negative correlation between l-DOPA dosage and neuropsychological tests of RAVLT-recognition and the Brixton test. This would suggest that PD-depressed patients drove the negative correlation between the PD-All group and the neuropsychological tests and indicates that in PD patients with depressive symptoms, higher doses of l-DOPA correlate with lower results on cognitive tests.

## Discussion

The present study analyzed the influences of depressive symptoms on-medication in PD and three major findings were determined: (A) PD-depressed patients received higher dosages of l-DOPA medication; (B) in PD-non-depressed patients l-DOPA dosage had a strong positive correlation with duration of disease, while in PD-depressed patients, this correlation was absent; (C) only l-DOPA had a strong positive correlation with depressive symptoms in PD-All group, specifically with the cognition and somatic components of the BDI-II scale.

### l-DOPA and depressive symptoms

One explanation of increased l-DOPA dosage administered to PD-depressed patients is the misdiagnosing of depression in PD patients, as it has been reported (13, 14). This could be due to specific differences between depression in PD and depression as a primary affective disorder (17–19, 68) but can also be due to often observed somatic symptoms of depression in PD patients without depression (20). Furthermore, multiple studies reported that depression in PD presents fewer dysphoric symptoms such as guilt and suicidal ideation and more somatic and cognitive symptoms, which additionally can be confused with Parkinsonism manifestations (17–19, 69, 70). Considering the above similarities and differences, some of the depressive symptoms might have been confused with PD aggravation and might have motivated the increase of l-DOPA dosages.

Strikingly, our results showed a positive correlation in the group of all PD patients only between l-DOPA and the BDI-II scale somatic and cognitive components. Previous studies also reported that depression in PD presents more somatic and cognitive symptoms (17–19, 69, 70). Nevertheless, this positive correlation reveals either that (1) an increase of l-DOPA dosage could worsen the depressive symptoms, or (2) due to impairment of BDI-II components, the dosages of l-DOPA have been increased. Considering that PD-depressed patients were administered significantly higher dosages of l-DOPA and these dosages did not correlate with duration of disease nor with UPDRS score in PD-depressed, it is more likely the first case scenario.

Increased l-DOPA dosage in PD-depressed and the strong influence of l-DOPA on depressive symptoms can be explained by the differential dopaminergic depletion of ventral and dorsal striatum in PD (42, 43). It has been shown that in the initial phase of PD, the dopamine in the dorsal striatum is severely depleted, while the ventral striatum is relatively intact. Thus, the dopamine treatment in this phase will improve the dorsal striatum functions (the motor symptoms), while the l-DOPA overdose in the ventral striatum will determine a dysregulation of functions, inducing depressive symptoms. Interestingly, the deep brain stimulation of nucleus accumbens (ventral striatum) has been shown to decrease depression in treatment-resistant depression (71).

Additionally, the dopamine overdose effect in the ventral striatum has been linked with cognition impairment in PD patients in our previous study (43) and, our present study also revealed some results with respect to cognition. The ANOVA analyses showed a difference between the two PD groups on the RAVLT tests and the correlation analysis showed a negative correlation between l-DOPA dosage and the cognitive neuropsychological tests of RAVLT. Extensive studies in rodents showed that nucleus accumbens receives afferent projections from the prefrontal cortex (72–74) and it is believed that dopamine influences nucleus accumbens activation by prefrontal cortex afferents (75–77). From this point of view, the treatment induced dopamine overdose in the ventral striatum can have an influence on cognitive function associated with the prefrontal cortex. Some studies also reported that antiparkinsonian medication might be associated with cognitive impairment (78–80).

It has been reported that the prefrontal cortex, nucleus accumbens, and basolateral amygdala are the main targets of the meso-cortical and meso-limbic dopamine systems and represent core structures at the interfaces of addiction (81). Several studies demonstrated that rhythmic interactions between prefrontal cortex, nucleus accumbens, and basolateral amygdala are central to cognitive functions such as learning and memory (82–84). These regions are the site of severe functional adaptations and homeostatic impairments following chronic drug exposure (85–87). Interestingly, patients with DDS exhibit enhanced l-DOPA-induced ventral striatum dopamine release (41), and DDS has been associated with depressive symptoms (39). Thus, the excessive medication usage in PD patients might be determined initially by the presence of depressive symptoms due to ventral striatum dysfunction and further PD patients develop addiction-like behavior and abuse the l-DOPA in order to stimulate an excessive dopamine release.

In our study, the dopaminergic agonists did not show any specific impact on depressive symptoms or cognitive tests in any of the groups and there was no difference between the groups with respect to agonists’ dosage. This would indicate that agonists have no relationship with depression. An additional support for this view is that agonists have been reported to be more responsible for disorders of impulse control, hallucinations, and compulsions (88, 89) while l-DOPA is more linked to anxiety and depression (25). Taken together, the results argue for the use of non-dopaminergic approaches for the treatment of depressive symptoms in PD, such as the use of selective serotonin reuptake inhibitors (90–92).

### l-DOPA and motor function

The present study also revealed a positive correlation between l-DOPA and the UPDRS scores in all PD patients. This would mean that increased UPDRS scores correlated with increased l-DOPA dosage. Further, only PD-non-depressed patients showed a statistically significant positive correlation between the rigidity sub-group of the UPDRS scale and the l-DOPA dosage, while the same results for the PD-depressed group were not statistically significant. This would imply that in PD-non-depressed patients, l-DOPA dosages were administered based on the worsening of the UPDRS score, while in PD-depressed the treatment was adjusted based on other considerations, possibly including the presence of depressive symptoms. On the other hand, previously it has been reported a significant positive correlation between depression and UPDRS motor scores, including tremor and akinesia (93). Additionally, depression has been shown to impair even more the motor symptoms in PD (94). Our study showed the absence of statistically significant differences between the two PD groups with respect to the UPDRS score. Thus, the possibility that l-DOPA was administered in PD-depressed patients based on non-motor considerations is to be considered, yet it is also possible that stronger complaints from patients about the motor symptoms might also be an argument for increasing the l-DOPA dosage.

### Limitations

The present study has several limitations. First, we used the median of the BDI-II score as a limit between the two PD groups (PD-depressed and PD-non-depressed) and the results might be biased. However, BDI-II scale is not a diagnostic tool and thus reveals only the presence of depressive symptoms and it is often used and has been validated in PD population. In our study, its utility was confirmed by the significant statistical difference between the depressed and non-depressed groups with respect to l-DOPA dosage and cognitive performance on several neuropsychological tests. Second, our groups have a relatively low number of subjects. Third, the interaction between l-DOPA and dopamine agonists has not been ruled out and the effect of combination of both drugs on depressive symptoms has not been accounted. Fourth, one cannot conclude necessarily that dopamine overdosage in the ventral striatum induces functional impairment in the prefrontal cortex and, respectively, cognitive impairment only due to connectivity relationship between the two areas. Nevertheless, this study is strongly confirming the data from previous reports, and brings additional emphasize on l-DOPA influence. More research is needed to confirm the direction of the correlation between higher l-DOPA dosage and depressive symptoms.

## Conclusion

In conclusion, the results of this study show that in PD patients, higher l-DOPA dosages correlate with worse depressive symptoms and possibly with decreased results in some cognitive performances. From this point of view, PD patients need to be better diagnosed with respect to depressive symptoms and need additional treatment adjustment when clinical manifestations of depression are present. This might be performed by reducing the l-DOPA dosage or by receiving treatment specific for depressive symptoms (such as selective serotonin reuptake inhibitors). Clinicians must be aware that dopaminergic drugs are not sufficient to alleviate depressive symptoms. Further longitudinal investigation is needed to confirm the present hypothesis.

## Conflict of Interest Statement

Clotilde Degroot, Alexandru Hanganu, Christophe Bedetti, Béatriz Mejia-Constain, Marie-Andrée Bruneau: none. Oury Monchi: grants: Canadian Institutes of Health Research, Parkinson Society Canada, Natural Sciences and Engineering Research Council of Canada, Fonds de la Recherche Québec (Santé); Anne-Louise Lafontaine: Advisory Boards: UCB, Novartis, AbbVie; Honoraria: UCB, Novartis, AbbVie, Teva; Sylvain Chouinard: Consultancies: UCB, Novartis, AbbVie, Teva; Advisory Boards: UCB, Novartis, AbbVie, Teva; Partnerships: UCB, Novartis, AbbVie, Teva; Honoraria: UCB, Novartis, AbbVie, Teva.
